# Adipose-Derived Mesenchymal Stem Cell Exosomes Attenuate Oxygen–Glucose Deprivation-Induced Cochlear Damage by Inducing Autophagy-Associated Signaling

**DOI:** 10.3390/ijms27146108

**Published:** 2026-07-08

**Authors:** Yi-Chun Lin, Yuan-Yung Lin, Hang-Kang Chen, Hsin-Chien Chen, Chih-Hung Wang, Chin-Mao Hung, Ai-Ho Liao, Cheng-Ping Shih

**Affiliations:** 1 Department of Otolaryngology-Head and Neck Surgery, Tri-Service General Hospital, National Defense Medical University, No. 325, Sec. 2, Chenggong Rd., Neihu District, Taipei 11490, Taiwan; 2 Department of Otolaryngology-Head and Neck Surgery, School of Medicine, College of Medicine, National Defense Medical University, Taipei 11490, Taiwan; 3 Department of Otolaryngology, Taipei City Hospital, Taipei 103212, Taiwan; 4 Institute of Preventive Medicine, National Defense Medical University, New Taipei City 237010, Taiwan; 5 Graduate Institute of Medical Sciences, College of Medicine, National Defense Medical University, Taipei 11490, Taiwan; 6 Graduate Institute of Biomedical Engineering, National Taiwan University of Science and Technology, Taipei 106335, Taiwan; 7 Department of Biomedical Engineering, National Defense Medical University, Taipei 11490, Taiwan

**Keywords:** adipose-derived mesenchymal stem cells, autophagy, cochlea, exosome, hair cell, ischemia, oxygen–glucose deprivation

## Abstract

Ischemia plays a critical role in the pathogenesis of sensorineural hearing loss through the induction of severe cochlear apoptosis and mitochondrial dysfunction. Exosomes derived from adipose-derived mesenchymal stem cells (ADMSC-Exo) have robust protective effects under non-otologic ischemic conditions. However, their otoprotective effects remain unclear. This study aimed to investigate the protective effects of human ADMSC-Exo against cochlear damage and mitochondrial dysfunction under oxygen–glucose deprivation (OGD), an in vitro and ex vivo model of cochlear ischemia. ADMSC-Exo attenuated OGD-induced cytotoxicity and apoptosis in HEI-OC1 cells and reduced the OGD-induced loss of cochlear hair cells in the organ of Corti explants. OGD caused a decrease in mitochondrial mass and mitochondrial membrane potential depolarization and impaired mitochondrial respiration in auditory cells. ADMSC-Exo preserved mitochondrial integrity and improved mitochondrial bioenergetic function following OGD exposure. These effects were accompanied by increased LC3-II conversion and formation of autolysosome-like structures and elevated expression of PINK1 and Parkin, indicating the activation of autophagy and mitophagy-related protective mechanisms. Importantly, 3-methyladenine, an autophagy inhibitor, attenuated the cytoprotective effect of ADMSC-Exo, supporting the involvement of autophagy in ADMSC-Exo-mediated protection. Collectively, these findings suggest that ADMSC-Exo protect against OGD-induced cochlear injury by promoting autophagy-associated mitochondrial protection.

## 1. Introduction

Cochlear ischemia plays a significant role in the pathogenesis of several otological disorders, such as noise trauma, presbycusis and ototoxicity [1]. It has also been proposed as a potential contributor to sudden sensorineural hearing loss of vascular etiology. The cochlea is an organ with high metabolic demand, and mammalian cochlear hair cells exhibit extremely limited regenerative capacity [2,3]. Therefore, the cochlea is vulnerable to ischemic injury. Previous studies have reported functional and morphological changes in the ischemic cochlea in animals [3,4,5,6,7,8,9,10,11]. Ischemia injury leads to a decrease in endocochlear potential, disruption of cochlear homeostasis and hearing threshold shifts [10,11]. The injury extensively affects various cell populations within the cochlea, with damage to cochlear hair cells and afferent neurons recognized as the primary lesions involved in ischemia-induced hearing loss [3,5]. Following cochlear ischemia of varying durations, different degrees of structural damage occur in hair cells and spiral ganglion neurons. These types of damage include organelle swelling, cellular edema, plasma membrane rupture, stereocilia disorganization, and apoptosis. In an in vitro model of cochlear ischemia, oxygen–glucose deprivation (OGD) lead to mitochondrial dysfunction, characterized by mitochondrial shrinkage and depolarization in the marginal cells of the cochlear lateral wall [6]. Ischemic injury induces the expression of various genes, including HIF-1α, Bcl-2, and NF-κB, as well as proinflammatory cytokines and the production of reactive oxygen species in the cochlea [4,5,6]. These findings indicate that impaired cochlear blood flow elicits an inflammatory response and oxidative stress, exacerbating cochlear dysfunction. Sudden sensorineural hearing loss, an otological emergency, often has an unknown etiology; however, vascular impairment of the cochlea is recognized as the most common cause of this condition [12]. Clinical data and animal model findings suggest a close association between microcirculatory dysfunction, oxidative stress, and sudden sensorineural hearing loss [2]. Currently, approximately one-third of patients do not regain their hearing after treatment [13]. Therefore, more effective treatments for cochlear ischemia require further investigation.

Mesenchymal stem/stromal cell (MSC)-based strategies have therefore been proposed as potential approaches for the treatment of inner ear injury because of their anti-inflammatory, antiapoptotic, antioxidative, and tissue-supportive effects [14,15,16,17]. However, direct stem cell transplantation for the inner ear remains challenging, including difficulty in targeting the cochlea because of the anatomical structure of the labyrinth, limited engraftment efficiency, and safety concerns related to uncontrolled cell behavior [14]. Accordingly, cell-free therapy based on MSC-derived exosomes is now considered a major mediator of stem cell-associated therapeutic effects [18]. Owing to their small size, exosomes can pass through the inner ear via the blood–labyrinth barrier or the round window membrane [19,20]. Therefore, exosomes can be efficiently delivered through systemic administration or the intratympanic approach. Previous studies have demonstrated that exosomes derived from bone marrow or umbilical cord MSCs are potential therapeutics for drug-induced ototoxicity, noise trauma and degeneration of the auditory nerve [18,21,22,23,24]. Exosomes derived from spiral ganglion progenitor cells and neural progenitor cells overexpressing microRNA-21 can alleviate hearing loss in mice following ischemia–reperfusion injury by inhibiting hair apoptosis and the inflammatory response [5,25]. Among the available adult stem cell sources, adipose-derived MSCs (ADMSCs) are particularly attractive because they are abundant, can be obtained with relatively low invasiveness, and possess strong paracrine activity in addition to multilineage differentiation potential [26]. In terms of autologous use, ADMSC-derived exosomes (ADMSC-Exo) are more readily available and show great promise for clinical applications [26]. However, the otoprotective effects of ADMSC-Exo remain unknown. The aim of the present study was to investigate whether human ADMSC-Exo can protect against cochlear damage using an OGD-induced cochlear ischemia model and to explore the effects of human ADMSC-Exo on mitochondrial function in OGD-exposed auditory cells.

## 2. Results

### 2.1. ADMSC-Exo Exert a Protective Effect Against OGD-Induced Loss of Cochlear Hair Cells

**Figure 1:**
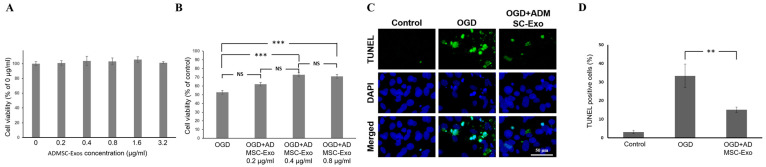
OGD-induced cytotoxicity and apoptosis in HEI-OC1 cells are attenuated by treatment with ADMSC-Exo. (**A**) Viability of HEI-OC1 cells after 48 h of treatment with various concentrations of ADMSC-Exo. n = 6 for each group. Data were analyzed using the Kruskal–Wallis test. No significant difference was detected among groups (overall *p* = 0.961). (**B**) Viability of HEI-OC1 cells treated with ADMSC-Exo under OGD conditions. The cells were treated with 0.2, 0.4, or 0.8 μg/mL ADMSC-Exo for 24 h during 24 h of OGD exposure. n = 16 for each group. Kruskal–Wallis test (*p* < 0.001) followed by Dunn’s post hoc test. OGD vs. OGD + ADMSC-Exo 0.4 μg/mL, *p* < 0.001; OGD vs. OGD + ADMSC-Exo 0.8 μg/mL, *p* < 0.001. NS, not significant; *** *p* < 0.001. (**C**) Representative image of the TUNEL assay in HEI-OC1 cells. Cells in the OGD + ADMSC-Exo group were treated with 0.4 μg/mL ADMSC-Exo for 24 h during OGD exposure. TUNEL-positive cells are highlighted in green, and DAPI-stained nuclei are indicated in blue. Scale bar: 50 μm. (**D**) Quantification of TUNEL-positive cells in different experimental groups. n = 5 for each group. Mann–Whitney U test for comparisons between the OGD and OGD + ADMSC-Exo groups, *p* = 0.008. ** *p* < 0.01.

ADMSC-Exo were characterized using nanoparticle tracking analysis, flow cytometry, and transmission electron microscopy (TEM) to confirm their particle size distribution, concentration, surface marker expression, and morphology (Supplementary Figure S1). Exosome markers, including CD9, CD63, and CD81, were detected on ADMSC-Exo. HEI-OC1 cells were treated with various concentrations of ADMSC-Exo for 48 h to evaluate the effect of ADMSC-Exo on cell viability (Figure 1A). Compared with the untreated control group, all the drug-treated groups maintained comparable levels of cell viability. These results suggest that the compound is well tolerated by the cells at concentrations up to 3.2 µg/mL, indicating low inherent cytotoxicity within this range. To evaluate the protective effects of ADMSC-Exo against OGD-induced injury, cells were treated with 0.2 μg/mL, 0.4 μg/mL, or 0.8 μg/mL ADMSC-Exo during 24 h of OGD exposure (Figure 1B). As expected, OGD alone markedly reduced cell viability to approximately 53% of that of the control group. Treatment with 0.2 μg/mL ADMSC-Exo slightly improved cell viability to 62.1%, whereas treatment with 0.4 μg/mL ADMSC-Exo or 0.8 μg/mL ADMSC-Exo resulted in more pronounced protective effects, with cell viability restoring to approximately 71–73%. There was a significant difference between the OGD group and the 0.4 μg/mL OGD + ADMSC-Exo group and the 0.8 μg/mL OGD + ADMSC-Exo group (*p* < 0.001). These results suggest that ADMSC-Exo protect HEI-OC1 cells against OGD-induced cytotoxicity (Supplementary Figure S2). The concentration of ADMSC-Exo was determined to be 0.4 μg/mL for subsequent experiments. TUNEL staining was performed to assess apoptosis in OGD-injured cells and in cells treated with ADM SC-Exo during OGD exposure (Figure 1C,D). In the control group, almost no TUNEL-positive cells were observed. In contrast, the OGD group showed a substantial increase in TUNEL-positive staining, indicating that severe apoptosis was induced by OGD. Cells treated with ADMSC-Exo exhibited markedly reduced OGD-induced TUNEL signals, with fewer apoptotic nuclei and more intact DAPI-stained nuclei. These results suggest that ADMSC-Exo inhibit OGD-induced cytotoxicity in auditory cells.

**Figure 2:**
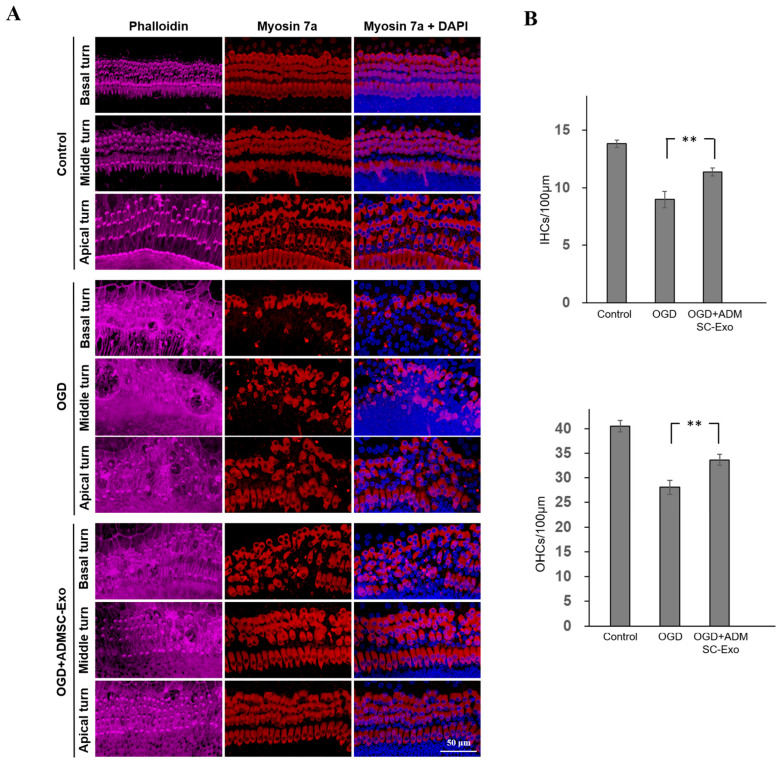
ADMSC-Exo ameliorate OGD-induced damage to the cochlea. (**A**) Representative images of organ of Corti explants are shown. The explants included those in the untreated control group, the OGD group (1.5 h OGD exposure), and the OGD + ADMSC-Exo group, which were treated with 0.4 μg/mL ADMSC-Exo for 1.5 h during OGD exposure. Phalloidin, stereociliary bundles (magenta); myosin 7a, cell bodies (red); DAPI, nuclei (blue). Scale bar: 50 μm. (**B**) Comparison of the number of cochlear hair cells in the different groups. n = 12 for each group. Mann–Whitney U test for comparisons between the OGD and OGD + ADMSC-Exo groups. ** *p* < 0.01.

Cochlear explants were treated with ADMSC-Exo during OGD exposure to evaluate the protective effects of ADMSC-Exo on cochlear hair cells damaged by OGD (Figure 2). Representative images show that hair cell damage was most pronounced in the OGD group across all cochlear turns. In contrast, the OGD + ADMSC-Exo group exhibited marked increases in cell survival in the basal, middle, and apical turns. As shown in Figure 2B, quantitative analysis further revealed significant differences in hair cell survival between the OGD and OGD + ADMSC-Exo groups. In terms of inner hair cells (IHCs), the mean survival count was significantly greater in the OGD + ADMSC-Exo group than in the OGD group (11.37 ± 0.36 vs. 8.98 ± 0.71, *p* = 0.002). Similarly, compared with that in the OGD group, the mean survival count of outer hair cells (OHCs) in the OGD + ADMSC-Exo group was significantly greater (33.65 ± 1.09 vs. 28.08 ± 1.40, *p* = 0.006). These findings indicate that compared with the OGD group, the OGD + ADMSC-Exo group exhibited significantly greater preservation of both IHC and OHC survival. ADMSC-Exo treatment strongly protected against OGD-induced injury to the cochlea.

### 2.2. ADMSC-Exo Ameliorate OGD-Induced Mitochondrial Dysfunction in Auditory Cells

**Figure 3:**
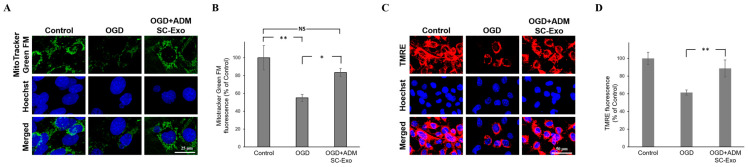
ADMSC-Exo improved OGD-induced mitochondrial damage in HEI-OC1 cells. (**A**) Representative confocal images of MitoTracker Green FM-stained HEI-OC1 cells after treatment with ADMSC-Exo and OGD exposure. Green indicates mitochondria; blue indicates Hoechst-stained nuclei. n = 4 for each group. Scale bar: 25 μm. (**B**) Quantification of MitoTracker Green FM fluorescence in different groups. n = 6 for each group. Kruskal–Wallis test (*p* = 0.003) followed by Dunn’s post hoc test. Control vs. OGD, *p* = 0.004; OGD vs. OGD + ADMSC-Exo, *p* = 0.024; Control vs. OGD + ADMSC-Exo, *p* = 1. NS, not significant; * *p* < 0.05, ** *p* < 0.01. (**C**) Representative images of TMRE staining in HEI-OC1 cells. TMRE (red); Hoechst-stained nuclei (blue). n = 4 for each group. Scale bar: 50 μm. (**D**) Comparison of TMRE fluorescence between groups with and without ADMSC-Exo treatment under OGD exposure. The mitochondrial membrane potential was evaluated using a TMRE ELISA kit. Mann–Whitney U test for comparisons between the OGD and OGD + ADMSC-Exo groups, *p* = 0.001. n = 8 for each group. ** *p* < 0.01.

MitoTracker Green FM staining was used to assess mitochondrial mass in control cells and OGD-injured cells, both with and without ADMSC-Exo treatment (Figure 3A,B). The OGD group showed a marked reduction in MitoTracker Green FM fluorescence, which was consistent with decreased mitochondrial content following OGD exposure. Compared with that in the OGD group, the fluorescence intensity in the OGD + ADMSC-Exo group increased, and the mitochondrial distribution in the OGD + ADMSC-Exo group improved. These results suggest that ADMSC-Exo can partially restore mitochondrial content in auditory cells following OGD injury. The mitochondrial membrane potential was compared among the three groups using tetramethylrhodamine ethyl ester (TMRE) staining (Figure 3C,D). The results revealed a significant decrease in fluorescence intensity in the OGD group compared with that in the control group. Compared with the OGD group, the OGD + ADMSC-Exo group exhibited more intense TMRE fluorescence (*p* = 0.001). These findings indicate that OGD causes mitochondrial depolarization in auditory cells, as evidenced by reduced TMRE fluorescence. Notably, ADMSC-Exo treatment partially restored the mitochondrial membrane potential and attenuated mitochondrial damage.

**Figure 4:**
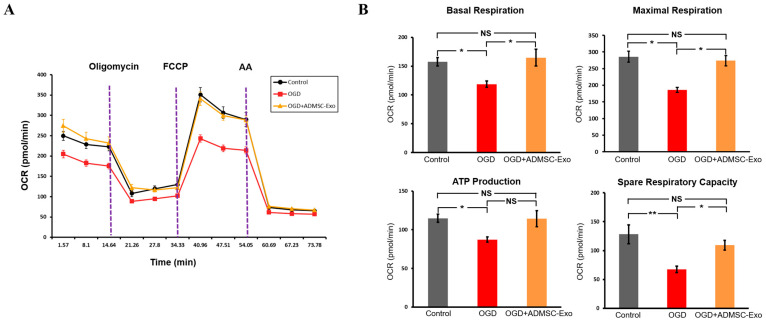
Effects of ADMSC-Exo on the mitochondrial bioenergetic profile of HEI-OC1 cells following OGD exposure. (**A**) Comparison of mitochondrial oxygen consumption rates (OCRs) among the control, OGD and OGD + ADMSC-Exo groups. The control group is represented by a black line, the OGD group by a red line, and the OGD + ADMSC-Exo group by an orange line. (**B**) Quantification of basal respiration, maximal respiration, ATP production, and spare respiratory capacity in the control, OGD and OGD + ADMSC-Exo groups. The data are presented as the means ± SEMs. n = 7 independent experiments per group. Kruskal–Wallis test followed by Dunn’s post hoc test. NS, not significant; * *p* < 0.05; ** *p* < 0.01.

OCR was assessed using the Seahorse XFp Cell Mito Stress Test to evaluate mitochondrial respiratory function among the control, ADMSC-Exo, OGD, and OGD + ADMSC-Exo groups (Figure 4). A comparison of bioenergetic profiles between the Control and OGD + ADMSC-Exo groups was shown in Supplementary Figure S3. The result indicates that ADMSC-Exo does not have a detrimental effect on mitochondrial bioenergetic function. Compared with the control group, the OGD group exhibited an overall reduction in OCR-related mitochondrial bioenergetic parameters, including basal respiration, maximal respiration, ATP production, and spare respiratory capacity, indicating impaired mitochondrial oxidative phosphorylation following injury. In contrast, compared with the OGD group, the OGD + ADMSC-Exo group exhibited marked recovery of the OCR profile, with increased basal respiration, maximal respiration, and spare respiratory capacity. Although ATP production showed an increasing trend after ADMSC-Exo treatment during OGD exposure, the difference between the OGD and OGD + ADMSC-Exo groups did not reach statistical significance (*p* = 0.075). Collectively, the results of the Seahorse OCR analysis demonstrated that OGD injury impaired mitochondrial bioenergetic function, as evidenced by reductions in basal respiration, maximal respiration, ATP production, and spare respiratory capacity. The OGD + ADMSC-Exo group effectively improved mitochondrial respiratory performance, particularly basal respiration, maximal respiration, and spare respiratory capacity. Although ATP production showed a nonsignificant trend toward recovery, the overall OCR profile suggested that ADMSC-Exo treatment preserved or restored mitochondrial function under OGD conditions.

### 2.3. ADMSC-Exo Protect Auditory Cells Against OGD-Induced Cytotoxicity by Activating Autophagy-Associated Signaling

**Figure 5:**
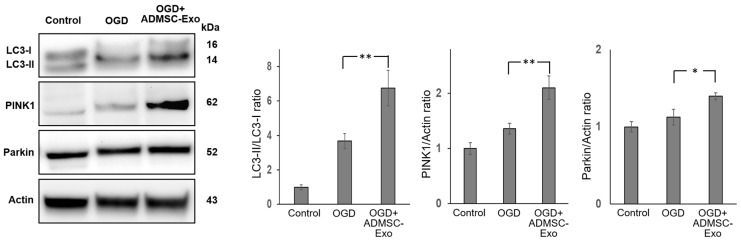
Western blot analysis of the protein expression levels of LC3-I, LC3-II, PINK1 and Parkin in HEI-OC1 cells following 24 h of OGD exposure and ADMSC-Exo treatment. n = 12 for each group. Quantitative data between OGD and OGD + ADMSC-Exo groups were analyzed using the Mann–Whitney U test, *p* = 0.003 in LC3-II/LC3-I ratio, *p* = 0.001 in PINK1/actin ratio, *p* = 0.033 in Parkin/actin ratio. * *p* < 0.05 and ** *p* < 0.01.

Autophagy plays a crucial role in protecting cochlear hair cells and is associated with cell survival in ischemia-damaged cochleae [27]. The relationship between autophagy and the protective effects of ADMSC-Exo was further investigated. Western blot analysis was conducted to explore the molecular mechanisms underlying changes in the expression levels of key autophagy- and mitophagy-related proteins—LC3, PINK1, and Parkin—across the three groups (Figure 5). Quantitative analysis revealed marked accumulation of LC3-II in the OGD + ADMSC-Exo group compared with the OGD group (*p* = 0.003), indicating that autophagy-associated signaling can be activated by ADMSC-Exo. PINK1 expression was significantly higher in the OGD + ADMSC-Exo group than in the OGD group (*p* = 0.001). Additionally, compared with that in the OGD group, Parkin expression in the OGD + ADMSC-Exo group was elevated (*p* = 0.033). These data suggest that ADMSC-Exo can lead to the activation of mitophagy, and that ADMSC-Exo may modulate both autophagy and mitophagy pathways, promoting mitochondrial homeostasis and cellular survival following ischemic injury in the cochlea.

**Figure 6:**
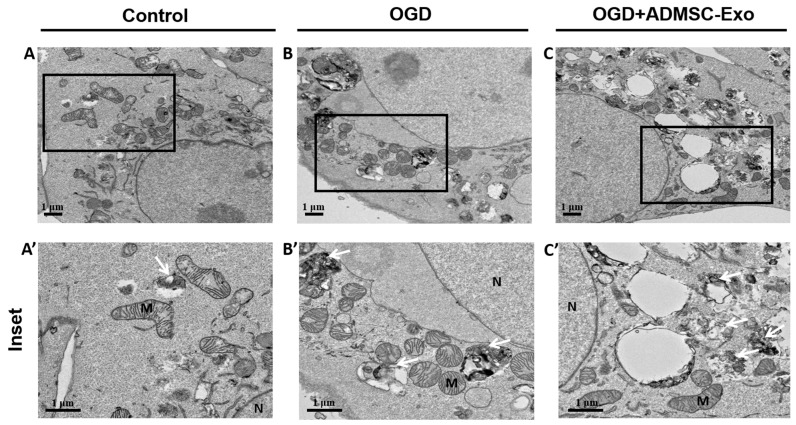
TEM images of HEI-OC1 cells after 24 h of OGD exposure and treatment with ADMSC-Exo. (**A**,**A′**) Control group. (**B**,**B′**) OGD group. (**C**,**C′**) OGD + ADMSC-Exo group. White arrows indicate autolysosome-like structures. M, mitochondria; N, nucleus. n = 3 for each group. Scale bar: 1 μm.

**Figure 7:**
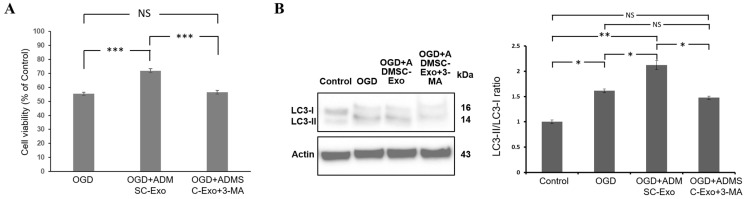
The therapeutic effect of ADMSC-Exo on OGD-induced cytotoxicity in HEI-OC1 cells is attenuated by 3-MA, an autophagy inhibitor. (**A**) Viability of HEI-OC1 cells cotreated with ADMSC-Exo and 3-MA under OGD conditions. Cells in the OGD + ADMSC-Exo + 3-MA group were cotreated with 3 mM 3-MA and 0.4 μg/mL ADMSC-Exo for 24 h during OGD exposure. n = 22 for each group. Kruskal–Wallis test (*p* < 0.001) followed by Dunn’s post hoc test. OGD vs. OGD + ADMSC-Exo, *p* < 0.001; OGD vs. OGD + ADMSC-Exo + 3-MA, *p* = 1; OGD + ADMSC-Exo vs. OGD + ADMSC-Exo + 3-MA, *p* < 0.001. NS, not significant; *** *p* < 0.001. (**B**) Western blot analysis of LC3-I and LC3-II in HEI-OC1 cells after OGD exposure and treatment with ADMSC-Exo and 3-MA at a concentration of 3 mM. n = 8 for each group. Quantitative data were analyzed using Kruskal–Wallis test (*p* < 0.001) followed by Dunn’s post hoc test. NS, not significant; * *p* < 0.05, ** *p* < 0.01.

TEM was used to evaluate ultrastructural changes associated with autophagy in HEI-OC1 cells across different treatment groups (Figure 6). In the control group, the cells exhibited a high density of mitochondria with well-preserved ultrastructural features. A limited number of autolysosome-like structures were observed in the cytoplasm, indicating relatively low autophagic activity in the control group. Compared with the control and OGD groups, OGD + ADMSC-Exo groups exhibited increased formation of autolysosome-like structures. These ultrastructural findings demonstrate that ADMSC-Exo treatment significantly enhances autophagic activity in OGD-exposed HEI-OC1 cells. The activation of autophagy-associated signaling by ADMSC-Exo is correlated with improved cellular integrity and increased survival following OGD injury. To further confirm the role of autophagy in the protective effects of ADMSC-Exo in our cellular model, cell viability was assessed following treatment with 3-methyladenine (3-MA), an autophagy inhibitor (Figure 7). The OGD + ADMSC-Exo group showed the highest cell viability, whereas the OGD group exhibited significantly lower cell viability than the OGD + ADMSC-Exo group (*p* < 0.001), demonstrating the protective effect of ADMSC-Exo against OGD injury. Additionally, cell viability was significantly lower in the OGD + ADMSC-Exo + 3-MA group than in the OGD + ADMSC-Exo group (*p* < 0.001). There was no significant difference in cell viability between the OGD + ADMSC-Exo + 3-MA and OGD groups, suggesting that inhibition of autophagy with 3-MA attenuated the protective effect of ADMSC-Exo observed in the OGD + ADMSC-Exo group. Taken together, these findings indicate that ADMSC-Exo attenuate OGD-induced injury to the cochlea by activating autophagy-associated signaling.

## 3. Discussion

For the first time, the present study demonstrates the protective potential of ADMSC-Exo in an OGD model. In non-otologic injury models, ADMSC-Exo also exhibit robust protective effects against ischemic and oxidative stress-related conditions, supporting the notion that the benefits of these treatments extend beyond a single organ system and reflect a broader reparative phenotype [26,28]. These exosomal cargos have been shown to regulate various signaling cascades involved in cell survival, inflammation, oxidative stress reduction, and autophagy modulation [26]. Previous studies have reported that ADMSC-Exo can promote autophagy to attenuate cardiac and cerebral ischemic injury [29,30,31,32]. The primary mechanisms through which ADMSC-Exo induce autophagy involve the mammalian target of rapamycin (mTOR) signaling pathway, the Sirtuin 1 (SIRT1) pathway, and the regulation of Ulk1 [32,33,34,35]. Our findings revealed that ADMSC-Exo increased LC3-II conversion and formation of autolysosome-like structures in auditory cells during OGD exposure, which simulated ischemic injury in vitro. These findings suggest that ADMSC-Exo attenuate cochlear injury in the OGD model by activating autophagy-associated signaling. The protective effect of ADMSC-Exo on OGD-exposed cells was attenuated by the administration of an autophagy inhibitor, indicating that autophagy activation plays a pivotal role in the protective effect of ADMSC-Exo against OGD-induced cochlear injury. Autophagy is a crucial cellular mechanism for maintaining hearing function and the development of the inner ear [27,36]. Following exposure to temporary threshold shift noise, autophagy is induced in OHCs to attenuate oxidative stress [37]. Potentiation of autophagy ameliorates noise-induced hearing loss and hair cell death [37]. Increased autophagy can reduce the accumulation of reactive oxygen species and apoptosis in the cochlea [36]. Yang et al. reported that autophagy is induced in the spiral ganglion of the cochlea following varying degrees of cochlear ischemia and reperfusion [38]. Excessive injury overwhelms the protective capacity of autophagy, leading to permanent hearing loss [38]. These findings suggest that autophagy plays an important role in the recovery from cochlear ischemia–reperfusion injury. SIRT1 activation and inhibition of mTORC1 signaling can promote cochlear hair cell survival by regulating autophagy [39,40]. Accordingly, in the present study, ADMSC-Exo induced autophagy-associated signaling to protect against cochlear OGD injury, possibly through SIRT1 activation or mTORC1 inhibition. Further investigations are needed to elucidate the mechanisms by which ADMSC-Exo induce autophagy in patients with cochlear OGD injury.

The effect of ischemia on mitochondrial function in auditory cells has rarely been investigated in the literature. The results of the present study demonstrated OGD-induced mitochondrial dysfunction in auditory cells and the therapeutic effect of ADMSC-Exo on impaired mitochondrial energy metabolism in OGD-exposed cells. OGD caused a decrease in mitochondrial integrity and mitochondrial membrane potential depolarization and impaired mitochondrial respiration in auditory cells. ADMSC-Exo treatment reversed the loss of mitochondrial mass and membrane potential. Seahorse extracellular flux analysis revealed that mitochondrial respiration in auditory cells was impaired by OGD. Compared with those in the control group, basal respiration and ATP production in the OGD group were reduced, indicating that OGD compromised mitochondrial activity under resting conditions. In contrast, compared with the OGD group, the OGD + ADMSC-Exo group exhibited higher basal respiration, suggesting that mitochondrial bioenergetic function was restored. The OGD group also showed marked reductions in maximal respiration and spare respiratory capacity, indicating limited mitochondrial reserve and a reduced ability to respond to increased energy demand. Both parameters improved in the OGD + ADMSC-Exo group compared with those in the OGD group, suggesting that ADMSC-Exo treatment preserved mitochondrial adaptability under stress conditions. Although ADMSC-Exo treatment restored basal respiration, maximal respiration, and spare respiratory capacity, ATP production did not significantly increase. This discrepancy may suggest that the treatment primarily improved the mitochondrial respiratory reserve and overall oxidative capacity, whereas ATP production may require a longer recovery period or a larger sample size to reach statistical significance. Taken together, these findings indicate that OGD injury impairs multiple aspects of mitochondrial bioenergetic function. ADMSC-Exo reversed these abnormalities and improved mitochondrial performance under both basal conditions and stress-induced conditions. Mitophagy, a form of selective autophagy that removes damaged mitochondria, has been shown to have a protective effect on cochlear hair cells [41]. In the present study, the expression levels of PINK1 and Parkin, two proteins related to mitophagy, were significantly greater in the OGD + ADMSC-Exo group than in the OGD group, suggesting that ADMSC-Exo activate mitophagy. The expression of PINK1 and Parkin decreases in various models of hearing loss [42]. Activation of the PINK1/Parkin-dependent pathway can ameliorate age-related changes in the cochlea, drug-induced ototoxicity, and noise trauma [42,43,44,45,46]. Our findings align with these observations, demonstrating that ADMSC-Exo can protect mitochondrial integrity and respiration against OGD injury, possibly by promoting the PINK1/Parkin-dependent mitophagy pathway. In MSC-derived exosomes, the cargos that activate PINK1/Parkin-related mitophagy signaling include miR-486-3p, miR-223-3p, miR-25-3p and GrpE-like 1 [47,48,49,50]. These cargos may contribute to the induction of the PINK1/Parkin pathway by ADMSC-Exo in the present study. Future studies are required to define the upstream ADMSC-Exo-derived regulators involved in cochlear ischemic injury.

These results highlight that ADMSC-Exo-based strategies may represent a promising approach for protecting cochlear cells from ischemic injury and underscore the importance of autophagy as a therapeutic target under such conditions. However, further studies are necessary to elucidate the specific molecular cargos responsible for autophagy induction and to optimize exosome dosage, administration routes, and delivery timing for in vivo applications. Future investigations employing omics profiling and in vivo animal models of ischemia will be essential to validate these findings and develop a mechanistic framework for therapeutic translation.

There are limitations in the present study. First, direct microscopic evidence of ADMSC-Exo uptake by HEI-OC1 cells was not obtained in the present study; therefore, whether the observed protective effects require direct vesicle internalization or involve indirect extracellular mechanisms remains to be determined. Second, 3-MA is not a fully specific autophagy inhibitor and may suppress class I PI3K-dependent signaling. The protective effect of ADMSC-Exo and the increased LC3-II conversion in auditory cells were inhibited by 3-MA. This suggests that ADMSC-Exo protects against OGD-induced cochlear injury partly by promoting autophagy-associated signaling. The role of class I PI3K in the protective effect of ADMSC-Exo will be evaluated further. Third, autophagic flux was not investigated in the present study. Increased LC3-II conversion may result from increased autophagosome formation or impaired autophagosome degradation. Autophagy activation plays a protective role in the cochlea [24,27]. Previous studies report that the blockage of autophagosome-lysosome degradation leads to apoptosis in auditory cells [51,52,53]. In the present study, ADMSC-Exo enhances LC3-II conversion during OGD exposure and exerts a protective effect against OGD-induced loss of auditory cells. Accordingly, ADMSC-Exo likely induces autophagy to protect against OGD-induced injury. Forth, Autolysosome-like structures can be detected on TEM. However, LC3 immuno-EM or double labeling with autophagy and lysosomal markers would provide more definitive confirmation of autolysosomes. Such experiments were not included in the present study. Fifth, there is the lack of in vivo validation. HEI-OC1 cells are a conditionally immortalized auditory cell line and therefore do not fully reproduce the phenotype, maturation status, electrophysiological properties, and cellular complexity of native cochlear hair cells. Similarly, organ of Corti explants prepared from neonatal mice are useful for evaluating cochlear cellular responses ex vivo, but they differ from the adult mature cochlea in developmental stage, tissue architecture, metabolic state, vulnerability to injury, and regenerative capacity. Further studies in animal models of ischemia will be essential to support our findings.

## 4. Materials and Methods

### 4.1. Cell Culture and In Vitro OGD Model

The HEI-OC1 mouse auditory cell line was generously provided by Dr. Federico Kalinec (House Ear Institute, Los Angeles, CA, USA). Cells were cultured in Dulbecco’s modified Eagle’s medium (DMEM; Gibco, Thermo Fisher Scientific, Waltham, MA, USA) supplemented with 10% fetal bovine serum (Thermo Fisher Scientific) and maintained at 33 °C in a humidified atmosphere containing 10% CO_2_. For assays, cells were seeded in 96-well plates at a density of 2–4 × 10^3^ cells per well and incubated overnight to allow for attachment. Human ADMSC-Exo were purchased from Immunostep S.L. (Salamanca, Spain). The reporting of ADMSC-Exo characterization was updated with reference to the MISEV2023 recommendations [54]. The ADMSC-Exo used in this study were commercially obtained from Immunostep S.L. According to the manufacturer’s technical information, the exosomes were derived from human ADMSCs and isolated by differential ultracentrifugation. The manufacturer reported characterization using nanoparticle tracking analysis, flow cytometry, Western blotting for EV-associated markers, and protein quantification. Endotoxin testing was not documented in the manufacturer’s technical datasheet available to us and was not independently performed in the present study. ADMSC-Exo were diluted in medium to the indicated concentrations and added to the cells. First, the cells were incubated with ADMSC-Exo for 48 h to evaluate the effect of ADMSC-Exo on cell survival. The in vitro OGD model was established as follows: cells were incubated in glucose-free DMEM and placed in a hypoxic chamber containing 1% O_2_ and 10% CO_2_ at 33 °C for 24 h. In the OGD + ADMSC-Exo group, the cells were treated with 0.4 μg/mL ADMSC-Exo for 24 h starting at the onset of OGD. Furthermore, 3-methyladenine (3-MA; Sigma-Aldrich, Burlington, MA, USA), an autophagy inhibitor, was used at a concentration of 3 mM to evaluate the role of autophagy in the protective effect of ADMSC-Exo against OGD-induced injury. The cells were collected for analysis after 24 h of treatment. The control cells were maintained under normoxic conditions in complete DMEM throughout the experiment.

### 4.2. Cochlear Explant Culture and Ex Vivo OGD Model

All animal procedures were conducted in accordance with the ethical standards and guidelines for laboratory animal care and use and were approved by the Institutional Animal Care and Use Committee (IACUC) of National Defense Medical University (NDMU), Taipei, Taiwan (Approval No. IACUC-23-026). The NDMU Animal Center is fully accredited by the Association for Assessment and Accreditation of Laboratory Animal Care International and provides standardized animal care and housing conditions in compliance with both institutional and international regulations. Thirty-six CBA/CaJ neonatal mice, including both male and female pups, were used in this study. One sample was collected from each animal. The animals were randomized into the control, OGD and OGD + ADMSC-Exo groups (control group (n = 12), OGD group (n = 12), OGD + ADMSC-Exo group (n = 12)). Their inner ears were collected at postnatal Day 3 following previously described procedures [55]. Cochlear explants were carefully dissected from the inner ears of mice under an Olympus SZX16 stereomicroscope (Olympus Corporation, Tokyo, Japan) in cold phosphate-buffered saline solution (PBS, Gibco, Waltham, MA, USA). The organ of Corti was isolated by removing the stria vascularis and spiral ligament and then transferred onto Cell-Tak-coated glass-bottom dishes (Ibidi, Grafelfing, Germany) to ensure firm adhesion. Explants were maintained in DMEM supplemented with 1% N2 supplement (Thermo Fisher Scientific) and 1% ampicillin at 37 °C in a humidified 5% CO_2_ incubator. After a 16 h stabilization period, the explants were divided into three groups: (1) the control group, which was cultured under normoxic conditions; (2) the OGD group, which was exposed to 1% hypoxic conditions (1% O_2_, 10% CO_2_) in glucose-free DMEM for 1.5 h; and (3) the OGD + ADMSC-Exo group, which underwent the same injury paradigm followed by cotreatment with 0.4 μg/mL ADMSC-Exo for 1.5 h. After OGD exposure and ADMSC-Exo treatment, explants were incubated in DMEM under normoxic conditions for 24 h. The tissues were then collected and immediately fixed in 4% paraformaldehyde for 30 min (Santa Cruz Biotechnology, Inc., Dallas, TX, USA).

### 4.3. Evaluation of Cell Viability and Apoptosis

Cell viability was determined using a WST-1 assay (Roche Diagnostics, Mannheim, Germany) according to the manufacturer’s instructions. Briefly, 10 µL of WST-1 reagent was added to each well containing 100 µL of culture medium and gently mixed. The plates were then incubated at 33 °C for 4 h. The absorbance was recorded at 450 nm with a 655 nm reference using an ELISA microplate reader (Synergy H4 Hybrid Reader, Agilent Technologies, Santa Clara, CA, USA). The cell viability is expressed as a percentage relative to that of the control group. A TUNEL assay (Merck KGaA, Darmstadt, Germany) was performed to evaluate apoptosis. The cells were incubated with the TUNEL reaction mixture at 37 °C for 1 h and then mounted in DAPI Fluoromount-G^®^ mounting medium (SouthernBiotech, Birmingham, AL, USA). The cell images were analyzed using an LSM 880 Zeiss confocal microscope (Carl Zeiss, Jena, Germany). TUNEL-positive cells were quantified by counting the number of TUNEL-positive nuclei and normalizing it to the total number of DAPI-stained nuclei in randomly selected fields.

### 4.4. Evaluation of Mitochondrial Mass and Membrane Potential

The mitochondrial mass was detected using MitoTracker Green FM (Thermo Fisher Scientific). The cells were incubated with MitoTracker Green FM dye for 30 min. After being washed, the cells were incubated with Hoechst 33342 (Thermo Fisher Scientific) for 5 min. The cells were imaged live using an LSM 880 Zeiss confocal microscope (Carl Zeiss). The mitochondrial membrane potential was assessed using TMRE (Cayman, Ann Arbor, MI, USA). The cells were incubated with TMRE (20 nM) in medium at 33 °C for 30 min in the dark and then analyzed using an ELISA microplate reader (Synergy H4 Hybrid Reader, Agilent Technologies, Santa Clara, CA, USA) with excitation/emission wavelengths of 530/580 nm. The relative TMRE fluorescence intensity was normalized to that of the control group and expressed as a percentage relative to that of the control group.

### 4.5. TEM

The cells were fixed at 4 °C overnight, followed by washing with cold PBS. The samples were then postfixed in 0.1 M cacodylate buffer for 2 h and washed three times with 0.1 M PBS for 15 min each. After dehydration through a graded ethanol series, the samples were infiltrated and embedded in Spurr’s resin. Polymerized resin blocks were cut into ultrathin slices approximately 90 nm thick using an ultramicrotome (Leica EM UC7, Leica Microsystems, Leica, Wetzlar, Germany). The sections were subsequently examined and imaged using a transmission electron microscope (FEI Tecnai 20 G2 S-Twin, FEI Company, Hillsboro, OR, USA).

### 4.6. Western Blotting

The protein concentration of the cell lysate was determined using a BCA assay (Thermo Fisher Scientific). Equal amounts of protein from the cell lysate were separated on mPAGE™ 4–12% Bis-Tris polyacrylamide gels (Merck KGaA, Darmstadt, Germany) and transferred to polyvinylidene difluoride membranes (Millipore, Billerica, MA, USA) via wet transfer (200 mA, 120 min, 4 °C). The membranes were blocked with BlockPRO™ blocking buffer (Energenesis Biomedical Co., Ltd., Taipei, Taiwan) for 1 h. The blots were incubated overnight at 4 °C with the following primary antibodies diluted in blocking buffer: LC3 (Cell Signaling; 1:1000), PINK1 (Proteintech, Rosemont, IL, USA; 1:500), Parkin (Proteintech; 1:1000), and the loading control pan-actin (Sigma-Aldrich, MA, USA; 1:1000). After being washed, the membranes were incubated with HRP-conjugated secondary antibodies (Cytiva, Marlborough, MA, USA; 1:10,000) for 1 h. The signals were developed using Immobilon^®^ Western Chemiluminescent HRP Substrate (ECL; Merck KGaA, Darmstadt, Germany) and imaged using a UVP ChemStudio Imager (Analytik Jena Co., Upland, CA, USA). More information about the antibodies is provided in the Supplementary Materials, Table S1.

### 4.7. Immunofluorescence Staining of the Cochlear Explants

The samples were incubated with anti-myosin VIIa antibody (Santa Cruz Biotechnology, Dallas, TX, USA) for 2 h. After being rinsed with PBS, the samples were incubated with Alexa Fluor™ 647 Phalloidin (1:500; Thermo Fisher Scientific) for 30 min. Finally, the samples were counterstained with DAPI (1:1000; Thermo Fisher Scientific) for 15 min. Images were acquired using an LSM 880 Zeiss confocal microscope (Carl Zeiss).

### 4.8. Seahorse XFp Cell Mito Stress Assay

Mitochondrial respiratory function was assessed using a Seahorse XFp extracellular flux analyzer with a Seahorse XFp Cell Mito Stress Test Kit (Agilent Technologies, Santa Clara, CA, USA; Cat. No. 103010-100). Briefly, cells were seeded in Seahorse XFp cell culture miniplates and incubated under standard culture conditions until the day of the assay. Prior to measurement, the culture medium was replaced with Seahorse XF assay medium (Agilent Technologies, Santa Clara, CA, USA; Cat. No. 1140121), and the cells were equilibrated in a non-CO_2_ incubator at 37 °C before analysis. The oxygen consumption rate (OCR) was measured under basal conditions and following sequential injections of mitochondrial stress reagents. The final working concentrations of the injected compounds were as follows: oligomycin, 1 μM; carbonyl cyanide-p-trifluoromethoxyphenylhydrazone (FCCP), 2 μM; and rotenone/antimycin A, 0.5 μM. Basal respiration, ATP respiration, maximal respiration, and spare respiratory capacity were calculated according to the manufacturer’s instructions. OCR values were normalized to the cell number and used for subsequent statistical analysis.

### 4.9. Statistical Analysis

Statistical differences among the groups were analyzed using the Mann–Whitney test for comparisons between two groups or the Kruskal–Wallis test followed by Dunn’s post hoc test for comparisons involving more than two groups. The results are presented as the means ± standard errors of the means (SEMs). Differences were considered to be statistically significant at *p* < 0.05.

## 5. Conclusions

The results of the present study demonstrate that human ADMSC-Exo provide significant protection against OGD-induced cellular injury in the cochlea. This cytoprotection and preservation of mitochondrial function are mediated primarily through the activation of autophagy and mitophagy-associated signaling. Future studies using appropriate animal models, dose optimization, and safety evaluation are required before clinical translation can be considered.

## Data Availability

The original contributions presented in this study are included in the article/Supplementary Materials. Further inquiries can be directed to the corresponding author.

## Supplementary Materials

The following supporting information can be downloaded at: https://www.mdpi.com/article/10.3390/ijms27146108/s1.

## Abbreviations

ADMSCs, adipose-derived mesenchymal stem/stromal cells; ADMSC-Exo, adipose-derived mesenchymal stem cells; FCCP, carbonyl cyanide-p-trifluoromethoxyphenylhydrazone; IACUC, the Institutional Animal Care and Use Committee; MSC, mesenchymal stem/stromal cell; mTOR, the mammalian target of rapamycin; NDMU, National Defense Medical University; OGD, oxygen–glucose deprivation; SIRT1, Sirtuin 1; TEM, transmission electron microscopy; TMRE, tetramethylrhodamine ethyl ester.
