# Supplementary material for: Adipose-Derived Mesenchymal Stem Cell Exosomes Attenuate Oxygen–Glucose Deprivation-Induced Cochlear Damage by Inducing Autophagy-Associated Signaling
Source: Int J Mol Sci. 2026 Jul 8;27(14):6108. doi: 10.3390/ijms27146108 (PMC13410168; doi:10.3390/ijms27146108)
Supplement: Supplementary file 1 [file ijms-27-06108-s001.zip › ijms-4381227-supplementary.pdf]

Figure S1. Confirmation and characterization of human ADMSC-Exo. (A) Particle size distribution of isolated ADMSC-Exo measured by nanoparticle tracking analysis. (B) Detection of the exosome markers CD9, CD63, and CD81 on ADMSC-Exo. ADMSC-Exo were stained with ExoBrite™ 405 EV Membrane Stain and analyzed using a CytoFLEX LX flow cytometer. (C) Transmission electron microscopy images of ADMSC-Exo at 20,000× (left) and 80,000× (right) magnification. (D) Confocal microscopy images of ADMSC-Exo labeled with anti-CD9 antibody and ExoBrite™ stain.

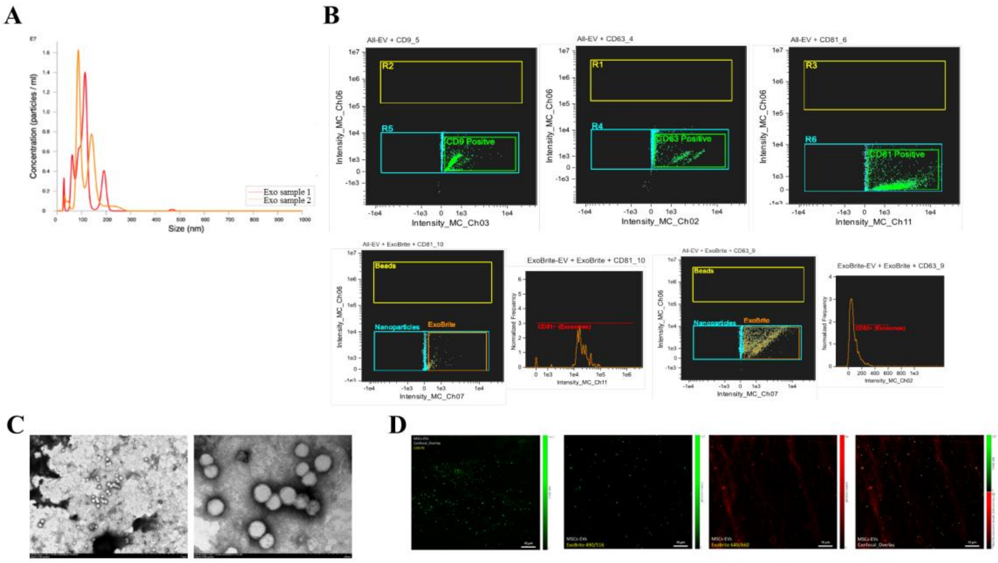

Figure S2. ADMSC-Exo treatment partially restores cell viability following OGD exposure. Cell viability was measured using the WST-1 assay and normalized to the control group. HEI-OC1 cells were exposed to OGD for 10 h or 24 h with or without ADMSC-Exo treatment. The cells were treated with 0.4 μg/mL ADMSC-Exo for 10 h during 10 h of OGD exposure (n = 5 for each group). The cells were treated with 0.4 μg/mL ADMSC-Exo for 24 h during 24 h of OGD exposure (n = 16 for each group). ADMSC-Exo significantly increased cell viability compared with the corresponding OGD-only groups at 10 h and 24 h of OGD exposure (OGD-10h vs. OGD-10h+ADMSC-Exo, $p = 0.029$; OGD-24h vs. OGD-24h+ADMSC-Exo, $p < 0.001$).

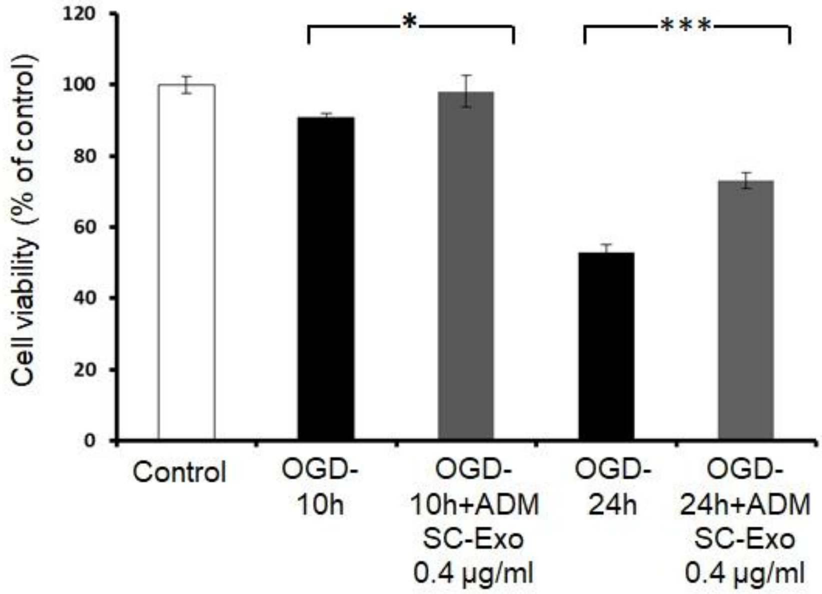

Figure S3. Effect of ADMSC-Exo on the mitochondrial biogenetic profile of HEI-OC1 cells. Comparison of the mitochondrial oxygen consumption rates (OCRs) in the Control and OGD+ADMSC-Exo groups. The Control group is represented by a black line, and the OGD+ADMSC-Exo group by a violet line. n = 4 independent experiments per group.

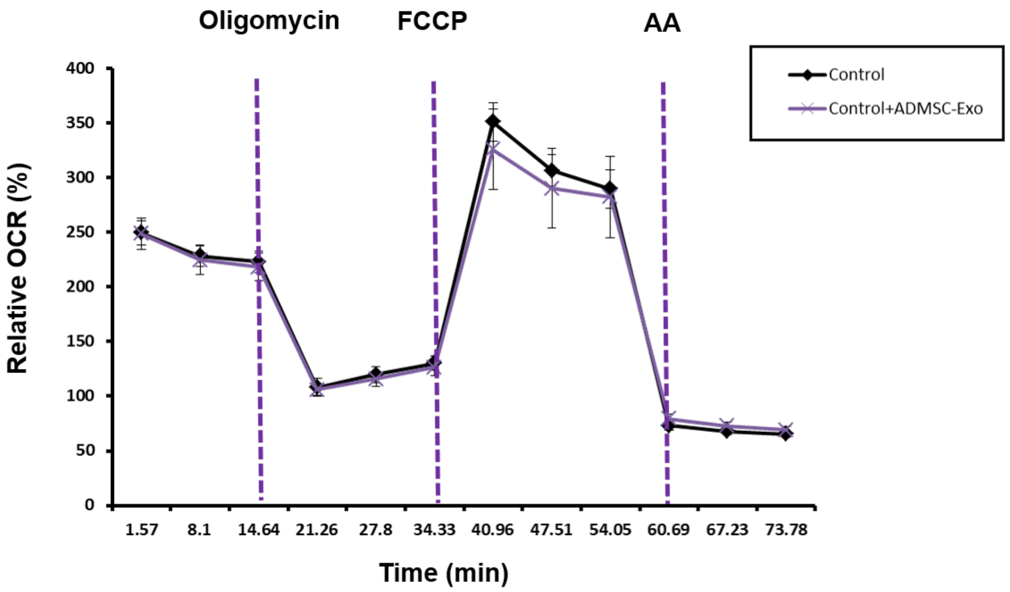

Table S1. Antibodies used in the present study

Target / Marker	Antibody type	Host / clonality	Manufacturer	Catalogue No.	RRID	Dilution	Application	Notes
LC3B	Primary antibody	Rabbit polyclonal	Cell Signaling Technology	2775	AB_915950	1:1000	Western blot	Autophagy marker
PINK1	Primary antibody	Rabbit polyclonal	Proteintech	23274-1-AP	AB_2879244	1:500	Western blot	Mitophagy-related marker
Parkin	Primary antibody	Rabbit polyclonal	Proteintech	14060-1-AP	AB_2878005	1:1000	Western blot	Mitophagy-related marker
Actin	Primary antibody	Mouse monoclonal, clone C4	Sigma-Aldrich	MAB1501	AB_2223041	1:1000	Western blot	Loading control
Myosin VIIa	Primary antibody	Mouse monoclonal, clone C-5	Santa Cruz Biotechnology	sc-74516	AB_2148626	1:100	IF	Hair cell marker
Mouse IgG	Fluorescent secondary antibody	Donkey anti-mouse IgG (H+L), highly cross-adsorbed	Thermo Fisher Scientific	A31570	AB_2536180	1:500	IF	Whole-mount IF
HRP-conjugated anti-rabbit IgG	HRP-conjugated secondary antibody	Donkey anti-rabbit IgG	Cytiva	NA934	AB_772206	1:10000	Western blot	Secondary antibody
HRP-conjugated anti-mouse IgG	HRP-conjugated secondary antibody	Sheep anti-mouse IgG	Cytiva	NA931	AB_772210	1:10000	Western blot	Secondary antibody
CD9	PE-conjugated anti-human CD9 antibody	Mouse monoclonal	Miltenyi Biotec	130-123-761	AB_2857632	1:50	Flow cytometry	Exosome marker
CD63	FITC-conjugated anti-human CD63 antibody	Mouse monoclonal	Miltenyi Biotec	130-123-673	AB_2811541	1:50	Flow cytometry	Exosome marker
CD81	APC-conjugated anti-human CD81 antibody, REAfinity™	Recombinant human IgG1 monoclonal	Miltenyi Biotec	130-119-787	AB_2751844	1:50	Flow cytometry	Exosome marker
